# Usefulness of color Doppler and strain elastography adjunctive to B-mode ultrasonography in the diagnosis of non-mass abnormalities of the breast: results of the BC-07 multicenter study of 385 cases

**DOI:** 10.1007/s10396-024-01485-1

**Published:** 2024-08-13

**Authors:** Toshitaka Okuno, Takanori Watanabe, Takuhiro Yamaguchi, Sachiyo Konno, Rie Takaki, Ryoji Watanabe, Kanako Ban, Koichi Hirokaga, Masahiko Tsuruoka, Takako Morita

**Affiliations:** 1 Department of Breast Surgery, Nishi-Kobe Medical Center, 7-5-1 Koji-dai, Nishi-ku, Kobe City, Kobe 651-2273 Japan; 2 Department of Breast Surgery, Sendai Medical Center, Sendai, Japan; 3 Division of Biostatistics, Tohoku University Graduate School of Medicine, Sendai, Japan; 4 Center of Medical Ultrasonics, Dokkyo Medical University Hospital, Shimotsuga-gun, Mibu, Japan; 5 Department of Clinical Laboratory, Hakuaikai Sagara Hospital, Kagoshima, Japan; 6 Department of Breast Surgery, Itoshima Medical Association Hospital, Itoshima, Japan; 7 Department of Breast Surgery, Yotsuya Medical Cube, Tokyo, Japan; 8 Department of Breast Surgery, Hyogo Cancer Center, Akashi, Japan; 9 Department of Radiology, Moriya Keiyu Hospital, Moriya, Japan; 10 Department of Breast Surgery, Nagoya Medical Center, Nagoya, Japan

**Keywords:** Doppler, Ultrasonography, Elasticity imaging techniques, Breast

## Abstract

**Purpose:**

The concept of non-mass abnormalities of the breast has been employed in Japan for approximately 20 years. Although B-mode findings are classified as non-mass abnormalities, the usefulness of adding color Doppler ultrasonography (US) and strain elastography to B-mode US is unclear. Therefore, we conducted a multicenter study (JABTS BC-07) to establish the diagnostic criteria for breast US, including color Doppler and elastography, for non-mass abnormalities of the breast and verify their diagnostic usefulness.

**Methods:**

We registered US images of non-mass abnormalities of the breast and their clinical and histopathological data from 13 institutions (202 malignant and 183 benign non-mass lesions). Furthermore, we evaluated the centralized image interpretation usefulness of the diagnostic criteria for B-mode and color Doppler US, as well as the sensitivity and specificity when color Doppler US and elastography were added to B-mode US.

**Results:**

Echogenic foci in the mammary gland (odds ratio 3.45, 95% confidence interval [CI] 1.92–6.19, p < 0.0001) and the configuration of internal solid components of the ducts (odds ratio 0.056, 95% CI 0.005–0.591, p < 0.0165) significantly differentiated benign and malignant non-mass abnormalities. The sensitivity of B-mode alone (83.7%) was significantly improved by adding color Doppler US (93.1%) (p = 0.0004); however, adding color Doppler US and elastography to B-mode US made no significant difference in either sensitivity or specificity.

**Conclusion:**

Although adding color Doppler US and elastography to B-mode US improved sensitivity, the diagnostic significance was limited. Therefore, a comprehensive diagnostic method comprising mammography and magnetic resonance imaging is warranted.

**Supplementary Information:**

The online version contains supplementary material available at 10.1007/s10396-024-01485-1.

## Introduction

With advances in ultrasound (US) diagnostic equipment and widespread adoption of breast cancer screening, the number of cases in which a non-mass lesion is detected in the breast is increasing. Non-mass abnormalities refer to lesions that do not meet the criteria for masses of the breast. Calcified lesions on mammography appear as hypoechoic areas within the mammary gland with echogenic foci, and cases of bloody nipple discharge appear as non-mass lesions such as intraductal echoes. However, no universal finding or terminology exists for these non-mass lesions [1, 2]. Moreover, conventional US reportedly has low diagnostic accuracy for non-mass lesions owing to overlapping of breast US features between malignant and benign non-mass lesions, such as fibrocystic changes, fibrosis, intraductal papilloma, ductal carcinoma in situ, and invasive lobular carcinomas [3–5].

**Fig. 1:**
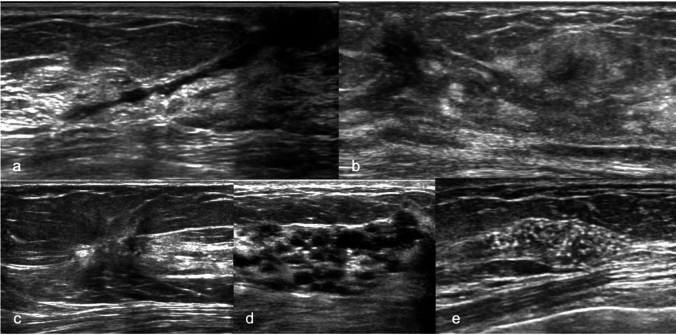
Classification of non-mass abnormalities of breast ultrasonography according to the Japanese Association of Breast and Thyroid Sonology (JABTS) and the Japan Society of Ultrasonics in Medicine (JSUM). **a** Ultrasound showing abnormalities of the ducts with irregular ductal caliber. The patient was diagnosed with invasive ductal carcinoma. **b** A case with a hypoechoic area in the mammary gland. After surgery, the lesion was diagnosed as ductal carcinoma in situ. **c** A case with a hypoechoic area in the mammary gland with architectural distortion, which was diagnosed as fibrocystic change. **d** Multiple small cysts are shown on B-mode ultrasound in a case of fibrocystic change. **e** Ultrasound showing echogenic foci without a hypoechoic area in the mammary gland, which was diagnosed as ductal carcinoma in situ

The Japan Association of Breast and Thyroid Sonology (JABTS) and the Japan Society of Ultrasonics in Medicine (JSUM) classify B-mode findings of non-mass abnormalities into the following four categories: hypoechoic areas in the mammary gland, abnormalities of the ducts, architectural distortion, multiple small cysts, and echogenic foci without a hypoechoic area [6–8] (Fig. 1). Echogenic foci, vascularity, and elasticity have been proposed as additional findings.

Color Doppler US reveals the vascularity of a tumor through visualization of the blood flow. However, it is not considered a standalone examination and is accompanied by B-mode US [9, 10]. Tissue elastography is another technique that can be used in breast US; two modes of tissue elastography, strain elastography (SE) and shear-wave elastography (SWE), are currently available. Previous studies reported that both SE and SWE improved the specificity in breast US diagnosis and reduced the need for biopsies [11–13].

We previously reported that the addition of both color Doppler US and SE to B-mode imaging improved the specificity without decreasing the sensitivity of the US diagnosis of breast masses [14]. This study aimed to clarify the usefulness of color Doppler US and SE adjunct to B-mode imaging and validate the JABTS and JSUM B-mode classification for non-mass abnormalities of the breast. We identified useful findings on color Doppler US and assessed the capability of color Doppler US and SE adjunct to B-mode imaging in distinguishing benign from malignant non-mass abnormalities of the breast.

## Materials and methods

The JABTS-BC 07 study was a multicenter observational study conducted along with CD-CONFIRM, a study verifying the utility of color Doppler US and elastography in the diagnosis of solid breast tumors, from April 2018 to March 2021 [14]. We enrolled women with pathologically confirmed non-mass abnormalities of the breast who underwent B-mode and color Doppler examinations and women with non-mass abnormalities of the breast with no significant change for more than 1 year. Non-mass abnormalities that remained unchanged for more than 1 year were considered benign. Clinical data and ultrasound images of non-mass abnormalities of the breast were collected, with the exclusion of lesions for which vacuum-assisted biopsies had previously been performed. In cases in which Real-time Tissue Elastography (FUJIFILM Medical Corporation) had been performed, images of elastography were also collected. We grouped bidirectional B-mode images, size measurement images, B-mode images of the same site on the contralateral side, B-mode videos, color Doppler videos, characteristic color Doppler images, and elastography images from cases in which Real-time Tissue Elastography had been performed.

The primary endpoints were the sensitivity, specificity, and accuracy of color Doppler US adjunct to B-mode US for non-mass abnormalities of the breast. The secondary endpoints were the validity of B-mode and color Doppler classifications and findings in the differential diagnosis between benign and malignant non-mass abnormalities of the breast and the sensitivity, specificity, and accuracy of color Doppler US and SE adjunct to B-mode US for non-mass abnormalities of the breast. We also performed an exploratory analysis comparing the distribution of vascularity between benign and malignant non-mass abnormalities by age.

### US examinations

A high-resolution US system with high-frequency (10 MHz and over) transducers was used in the study. The velocity range of the color Doppler was initially set to 3–4 cm/s. The velocity range, color scan area, and color gain were adjusted for vascularity. The subcutaneous fat and pectoral muscle layers were included in the region of interest of SE.

### Study registration

This study was registered with the University Hospital Medical Information Network, Japan (No. UMIN000032299).

### B-mode US diagnostic criteria for non-mass abnormalities of the breast

B-mode US images in this study were evaluated according to the criteria of non-mass abnormalities in the JABTS guidelines, location and distribution of the lesions, and echogenic foci as additional findings. Bilateral location or diffuse distribution indicate benign nature, while unilateral location and regional or quadrant distribution indicate malignant nature. The presence of echogenic foci in the hypoechoic area in the mammary gland suggests advancement of malignancy [6, 15].

### Color Doppler US diagnostic criteria for non-mass abnormalities of the breast

**Fig. 2:**
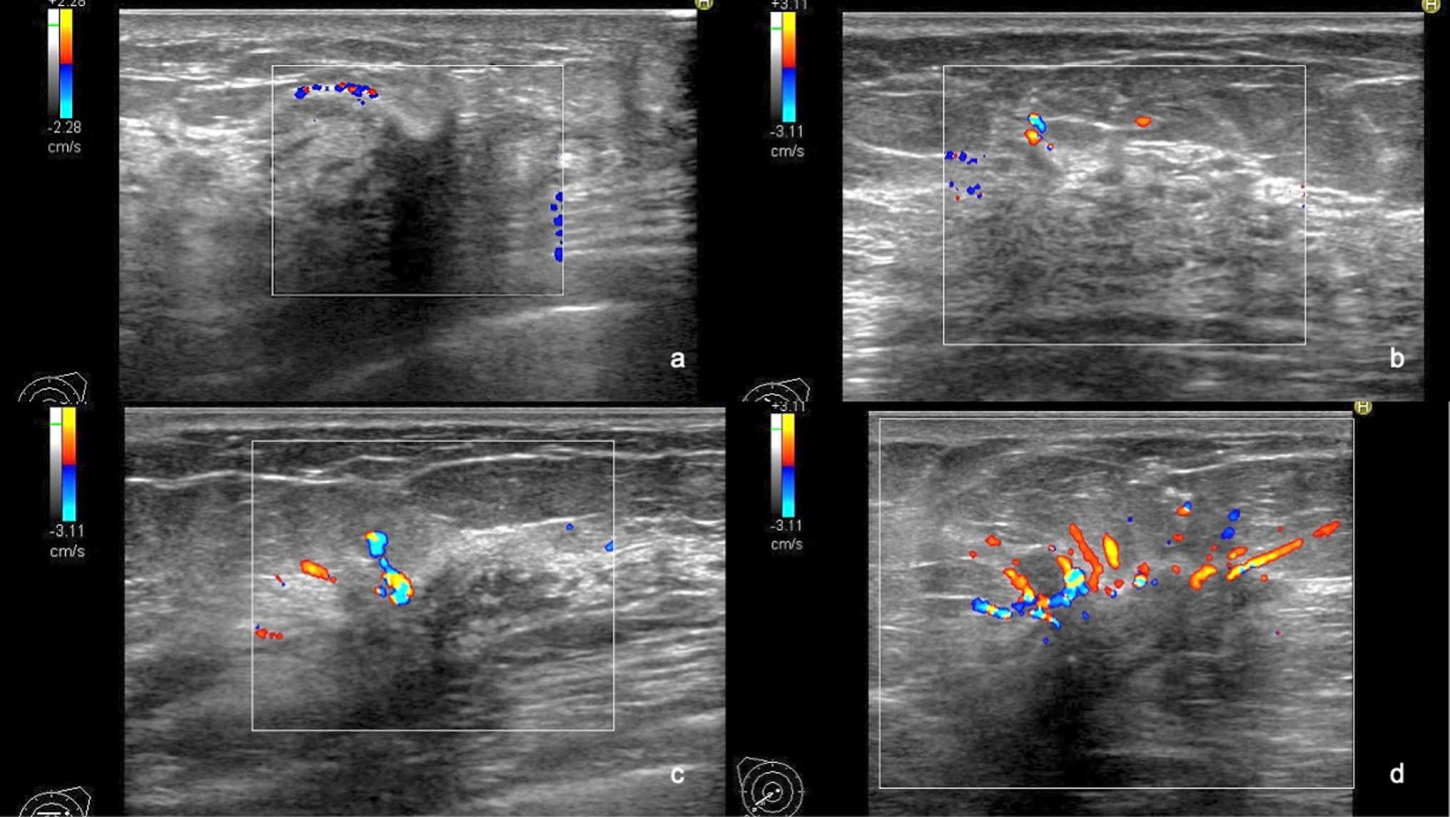
Evaluation of the vascularity of non-mass abnormalities of the breast on color Doppler ultrasonography. **a** This case with a surgical scar showed no vascularity and was assessed as vascularity (–). **b** Scant vascularity was demonstrated in a hypoechoic area within the mammary gland. Vascularity (1 +) was scored for this case with fibrocystic change. **c** This case of invasive ductal carcinoma with a predominant intraductal component showed moderate vascularity and was assessed as vascularity (2 +). **d** Abundant vascularity was demonstrated in a hypoechoic area within the mammary gland. Vascularity (3 +) was scored for this case of invasive ductal carcinoma

**Fig. 3:**
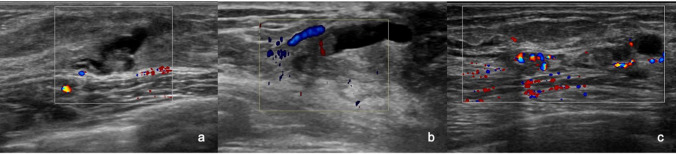
Vascularity of the solid component in the mammary ducts. **a** This case of papillary apocrine metaplasia in mammary duct ectasia showed no vascularity and was assessed as vascularity (–). **b** A single afferent blood flow into the solid component was demonstrated in this case of intraductal papilloma, which was assessed as vascularity (1 +). **c** This case of ductal carcinoma in situ showed multiple afferent blood supplies to the solid components in the duct. It was evaluated as vascularity (2 +)

To our knowledge, there are no well-known definitions or diagnostic criteria for color Doppler US of non-mass abnormalities of the breast. Therefore, the Terminology and Diagnostic Criteria Committee of the JABTS has developed definitions and diagnostic criteria for color Doppler US. Vascularity was evaluated subjectively according to the criteria of breast masses in the CD-CONFIRM study, i.e., according to a 4-point scale: 0, avascular; (1 +), hypo-vascular; (2 +), moderately vascular; and (3 +), hyper-vascular (Fig. 2). Penetrating or plunging blood flow in hypoechoic areas of the mammary gland and lesions with architectural distortions were suspected to be malignant. In ducts with internal solid components, a single afferent blood flow into the solid component implied a benign lesion, and multiple blood flows were suspected to be indicative of malignancy (Fig. 3).

### Evaluation for elastography images of non-mass abnormalities of the breast

**Fig. 4:**
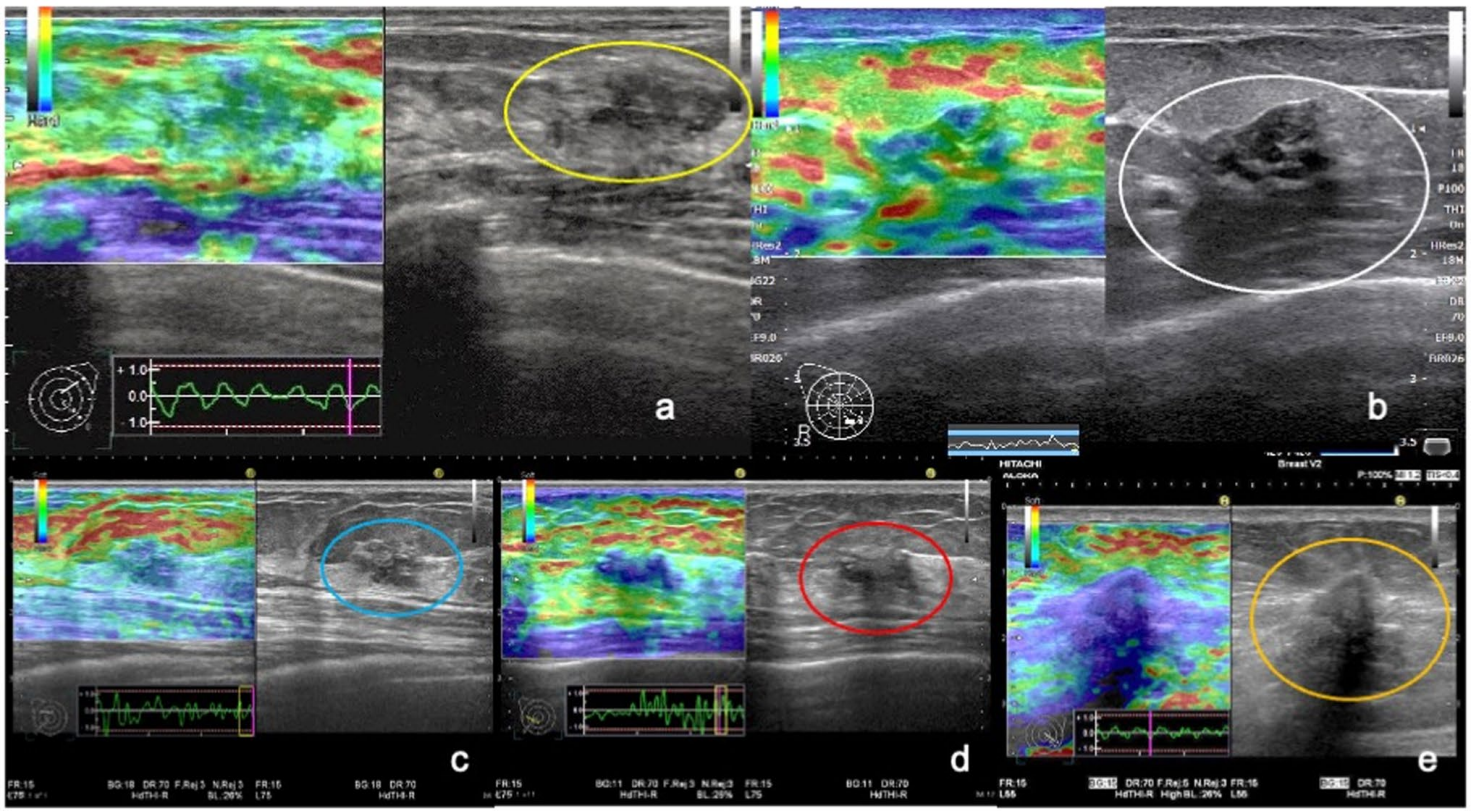
Diagnostic criteria for non-mass abnormalities on Real-time Tissue Elastography. **a** Elasticity score 1. The entire area of the lesion (yellow circle) displays green in a case with fibrocystic change, indicating soft tissue. **b** Elasticity score 2. The area of the lesion (white circle) displays green and blue, but predominantly green, in a case with fibrocystic change. **c** Elasticity score 3. The area of the lesion (blue circle) displays green and blue, but predominantly blue, in a case of ductal carcinoma in situ*.* **d** Elasticity score 4. The entire area of the lesion (red circle) displays blue in a case of ductal carcinoma in situ*,* indicating hard tissue. **e** Elasticity score 5. The extensive area of the lesion (orange circle) displays blue in a case of invasive ductal carcinoma

Various elastography methods exist, each with its own evaluation method. Real-time Tissue Elastography is widely used in Japan; thus, most of the elastography procedures registered in this study employed this application. Breast masses examined using Real-time Tissue Elastography are widely evaluated using the Tsukuba elasticity score in Japan [16]. We formulated the diagnostic criteria for non-mass abnormalities of the breast based on Real-time Tissue Elastography as follows: score 1, the entire area of the lesion displays green, indicating soft tissue; score 2, the area of the lesion displays both green and blue, but predominantly green; score 3, the area of the lesion displays both green and blue, but predominantly blue; score 4, the entire area of the lesion displays blue, indicating hard tissue; and score 5, extensive area of the lesion displays blue (Fig. 4).

### Japanese diagnostic categories

In Japan, the following diagnostic categories are used: Category 1, normal; Category 2, benign; Category 3a, probably benign (surveillance is recommended); Category 3b, probably benign (biopsy is recommended); Category 4, suspected malignancy; and Category 5, malignant. Japanese Category 3b, 4, and 5 correspond to the breast imaging-reporting and data system (BI-RADS) Category 4A, 4B and 4C, and 5 respectively (Supplementary Table 1).

### Centralized image interpretation

The centralized image interpretation committee comprised 16 breast US specialists, including one general physician, 10 breast surgeons, and five US technologists. They all had over 15 years of experience in breast US examinations, and the five US technologists were certified sonographers of JSUM. The images were interpreted without access to medical or background information other than the patient’s age. A remote US image interpretation system, developed for JABTS BC-07 and CD-CONFIRM by Zenbe Corporation, was used. By employing this system, image readers could evaluate the data on their computers at their convenience. First, the B-mode images were evaluated, and the B-mode category was subsequently determined. Color Doppler images were evaluated, and categorization combining B-mode and color Doppler imaging was performed. Cases with available elastographic images were also evaluated, and a category combining B-mode, color Doppler, and elastographic findings was assessed. For vascularity (–), we added -1 to the B-mode category, and for vascularity (2 +) and (3 +), we added + 1 to the B-mode category. Furthermore, elasticity scores of 4 and 5 were given a category with + 1 added to the B mode category.

### Data collection and statistical analysis

Data collection and statistical analyses were performed at the Clinical Research Data Center of Tohoku University Hospital. Clinical data and US findings were registered using an electrical data capture system built by Viedoc Japan, and US images were collected using network-attached storage via the Internet. Statistical analyses were performed using SAS software version 9.4. (SAS Institute Inc., Cary, NC, USA). For statistical analysis of sensitivity and specificity, Japanese categories 2 and 3a were considered negative, i.e., benign, whereas categories 3b, 4, and 5 (biopsy is recommended) were considered positive, i.e., malignant. The McNemar test was used to compare B-mode imaging alone; the combination of B-mode imaging and color Doppler imaging; and the combination of B-mode imaging, color Doppler imaging, and elastography. Univariate and multivariate logistic regression analyses were used to estimate odds ratios for the association between B-mode, color Doppler, and elastography findings of malignant non-mass abnormalities. *P* values less than 0.05 were considered statistically significant.

## Results

### Study sample selection

A total of 399 non-mass abnormalities were identified in 12 institutions. Of these, 11 were excluded owing to inadequate B-mode or color Doppler imaging. Three non-mass abnormalities with unknown histopathology were excluded. We ultimately analyzed 385 non-mass abnormalities, comprising 202 malignant and 183 benign lesions. Of the 385 non-mass abnormalities, 237 were examined simultaneously using Real-time Tissue Elastography (Supplementary Fig. 1).

The median age of the patients with 202 malignant and 183 benign non-mass abnormalities was 55 (interquartile range [IQR]: 47–67) years and 48 (IQR: 43–60) years, respectively. The histopathological results of the 385 non-mass abnormalities are summarized in Table 1. Among the benign lesions, 110 were pathologically confirmed and 73 were 1-year flow-up lesions.

**Table 1:** Histopathological results (N = 385)

Malignant lesions (N = 202)	
Noninvasive ductal carcinoma	94
Noninvasive lobular carcinoma	2
Microinvasive carcinoma	11
Invasive ductal carcinoma	82
Invasive lobular carcinoma	10
Apocrine carcinoma	1
Others	2
Benign lesions (N = 183)	
Fibrocystic change	51
Fibrous disease	8
Intraductal papilloma	8
Fibroadenoma	5
Radial scar and complex sclerosing lesion	3
Duct ectasia	2
Mastitis	1
Others^a^	32
Unknown (observation)^b^	73

^a^Benign lesions of unknown histological type

^b^One-year observation completed

The 385 non-mass abnormalities included 272 hypoechoic areas in the mammary gland (155 malignant and 117 benign) and 62 abnormalities of the ducts (40 malignant and 22 benign), including 54 lesions with internal solid components, 22 lesions with architectural distortion (8 malignant and 14 benign), 22 lesions with multiple small cysts (12 malignant and 10 benign), and seven malignant lesions of echogenic foci without a hypoechoic area. The 202 malignant lesions included 94 noninvasive ductal carcinomas, 82 invasive ductal carcinomas, 10 invasive lobular carcinomas, and others. The 183 benign lesions included 51 fibrocystic changes, eight cases of fibrous disease, eight cases of intraductal papilloma, and others. Seventy-three cases with benign lesions had unknown histological results and have been observed (Table 1).

### Sensitivity, specificity, and accuracy of the combination of B-mode and color Doppler US

The sensitivity, specificity, and accuracy of B-mode alone were 83.7%, 32.8%, and 59.5%, respectively. The sensitivity, specificity, and accuracy of B-mode plus color Doppler US were 93.1%, 29.0%, and 62.6%, respectively. The sensitivity of B-mode alone was significantly improved by the addition of color Doppler imaging (p = 0.0004). The specificity of B-mode alone was decreased by the addition of color Doppler US; however, the difference was not statistically significant. The accuracy of B-mode alone was improved by adding color Doppler US; however, the difference was not statistically significant (Table 2).

**Table 2:** Sensitivity and specificity of B-mode US alone (B), combination of B + color Doppler (CD), and combination of B + CD + elastography (E)

	B alone	B + CD	*p value*	B + CD + E	*p *value
Color Doppler analysis (N = 385)					
Sensitivity	83.7%	93.1%	0.0004*		
Specificity	32.8%	29.0%	0.2858*		
Accuracy	59.5%	62.6%	0.1573		
Elastography analysis (N = 237)					
Sensitivity	84.6%	95.9%		89.4%	0.1088*
Specificity	34.2%	26.3%		32.5%	0.7237*
Accuracy	60.3%	62.4%		62.0%	0.5553

^*^Compared with B-mode alone (McNemar’s test)

### Sensitivity, specificity, and accuracy of the combination of B-mode US, color Doppler US, and elastography

The sensitivity, specificity, and accuracy of B-mode US alone were 84.6%, 34.2%, and 60.3% respectively. The sensitivity, specificity and accuracy of B-mode US + color Doppler US + elastography were 89.4%, 32.5%, and 62.0% respectively. The sensitivity of B-mode US alone was improved by adding color Doppler US and elastography, but the difference was not significant (p = 0.1088). The specificity and accuracy of B-mode US alone was not altered by the addition of color Doppler US and elastography (Table 2).

### B-mode features and benign/malignant differential diagnosis

We studied the B-mode features and benign/malignant differential diagnoses in each subtype of non-mass abnormalities. Considering the 272 lesions of hypoechoic areas in the mammary gland, the presence of echogenic foci significantly differentiated benign and malignant non-mass abnormalities (odds ratio 3.45, 95% CI 1.92–6.19, p < 0.0001). Distributions of hypoechoic areas in the mammary gland were not statistically significant findings (odds ratio 1.52, 95% CI 0.85–2.74, p = 0.159) (Table 3a).

**Table 3:** B-mode features and benign/malignant differential diagnosis

Variable	Findings	Odds ratio	95% CI	*p* value
a. Distribution and echogenic foci in the hypoechoic areas of the mammary glands (*n* = 272)				
Distribution	Segmental vs. Focal	1.52	0.85–2.74	0.159
Echogenic foci	Present vs. Absent	3.45	1.92–6.19	< 0.0001
b. Configuration and distribution of the internal solid component of the ducts (*n* = 54)				
Configuration of internal solid component	Narrow-based vs. Broad-based	0.0560	0.005–0.591	< 0.0165
Distribution	Multiple vs. Solitary	0.579	0.125–2.69	0.485

*CI* confidence interval

Considering the 54 internal solid components of the ducts, the type of configuration significantly differentiated benign and malignant non-mass abnormalities (odds ratio 0.056, 95% CI 0.005–0.591, p < 0.0165). Distributions of internal solid components of the ducts were not statistically significant findings (odds ratio 0.579, 95% CI 0.125–2.69, p = 0.485) (Table 3b).

Distributions of the 22 multiple small cysts were not statistically significant findings (clustered vs. scattered: odds ratio 6.00, 95% CI 0.463–77.7; clustered vs. segmental: odds ratio 2.00, 95% CI 0.271–14.8; p = 0.338).

Neither the distributions of the 22 architectural distortions nor those of the seven lesions of echogenic foci without a hypoechoic area were statistically significant findings for distinguishing benign and malignant tumors.

### Color Doppler features and benign/malignant differential diagnosis

Vascularity was a statistically significant finding for distinguishing between 117 benign and 155 malignant hypoechoic areas in the mammary glands [(2 +)/(3 +) vs. (–)/(1 +), odds ratio 7.84, 95% CI 4.55–13.5, p < 0.0001]. Penetrating blood flows were also statistically significant findings for distinguishing between benign and malignant tumors (odds ratio 7.46, 95% CI 4.33–12.8, p < 0.0001) (Table 4a).

**Table 4:** Color Doppler features and benign/malignant differential diagnosis

Variable	Findings	Odds ratio	95% CI	*p* value
a. Vascularity and penetrating blood flows in the hypoechoic area of the mammary glands (*n* = 272)				
Vascularity	(2 +)/(3 +) vs. (1 +)/(–)	7.84	4.55–13.5	< 0.0001
Penetrating blood flow	Present vs. Absent	7.46	4.33–12.8	< 0.0001
b. Vascularity and afferent blood flow into the internal solid components of the ducts (*n* = 54)				
Vascularity	(2 +)/(3 +) vs. (1 +)/(–)	1.62	0.57–4.63	0.36
A single vascular stalk	Present vs. Absent	0.44	0.14–1.38	0.154
Multiple blood flows	Present vs. Absent	2.57	0.81–8.13	0.10

*CI* confidence interval

The vascularity of blood flow into an internal solid component of the ducts was not a statistically significant finding for distinguishing between 35 benign and 19 malignant lesions [(2 +)/(3 +) vs. (–)/(1 +), odds ratio 1.62, 95% CI 0.57–4.63, p = 0.36] (Table 4b). A single vascular stalk into an internal solid component of the ducts (present vs. absent, odds ratio 0.44, 95% CI 0.14–1.38, p = 0.154) and multiple blood flows (present vs. absent, odds ratio 2.57, 95% CI 0.81–8.13, p = 0.10) (n = 54) were not significantly different between benign and malignant tumors (Table 4b).

### SE features and benign/malignant differential diagnosis

Elasticity scores were significant findings for distinguishing between 70 benign and 92 malignant non-mass abnormalities (elasticity score 3/4/5 vs. elasticity score 1/2, odds ratio 4.49, 95% CI 2.20–9.16, p < 0.0001) (Table 5).

**Table 5:** Strain elastography features and benign/malignant differential diagnosis of the hypoechoic area in the mammary gland (*n* = 162)

Variable	Findings	Odds ratio	95% CI	*p* value
Elasticity score	Score 3/4/5 vs. Score 1/2	4.49	2.20–9.16	< 0.0001

*CI* confidence interval

### Distribution of vascularity by age between benign and malignant non-mass abnormalities

Because we did not collect data on menopausal status, we used a cutoff of 55 years, which is generally considered the age of menopause. The distribution of vascularity of malignant non-mass abnormalities in patients younger than 55 years did not differ from those in patients 55 years and older (p = 0.7361). In contrast, among benign non-mass abnormalities, patients younger than 55 years of age tended to have more lesions with high vascularity than those 55 years of age and older (p = 0.0144) (Supplementary Fig. 2).

## Discussion

### Terminology and classification of non-mass abnormalities on breast US

The incidence of breast cancer, which is the most common cancer in women, is increasing worldwide. Breast tumors are often discovered as breast masses on imaging; however, when accompanied by bloody nipple discharge or detected as calcification on mammography performed for screening purposes, they do not appear as a mass, and in many cases, they appear as non-mass abnormalities of the breast. Non-mass lesions are defined in the ACR BI-RADS breast magnetic resonance imaging (MRI) lexicon [17], but not in ultrasound imaging.

In Japan, JABTS and JSUM previously used the word “lesion,” which may be more popular worldwide, but “lesion” indicates disease. However, many non-mass lesions include aberrations from the normal that are not diseases. Therefore, JABTS and JSUM decided to use the term non-mass “abnormalities” based on the changes on images. After two revisions, the current terms were mainly categorized as hypoechoic areas in the mammary gland, ductal abnormalities, architectural distortion, multiple small cysts, and echogenic foci without a hypoechoic area [15]. Histological types presenting as non-mass abnormalities in the breast comprise a wide range of malignancies, including ductal carcinoma in situ, invasive lobular carcinomas, and invasive ductal carcinomas, and various benign lesions, such as fibrocystic change, sclerosing adenosis, and intraductal papilloma [18]. Thus, the JABTS taxonomy is too complex to accommodate the diagnosis of its diverse histology.

Many studies have classified non-mass lesions as ductal abnormalities, architectural distortions, or calcifications on US. It has also been reported that the diagnostic ability for non-mass abnormalities is not as satisfactory as that for masses, with fair sensitivity and poor specificity [1–5]. This is attributed to the overlap of a few US findings between low-grade ductal carcinomas in situ and benign diseases. The addition of color Doppler US or elastography to classical B-mode imaging reportedly improves the diagnostic specificity [19–22]. In the JABTS guidelines, calcification, vascularity, and elasticity are considered additional findings that supplement the B-mode findings [15].

### B-mode US, color Doppler US, and elastography of various non-mass abnormalities of the breast

**Fig. 5:**
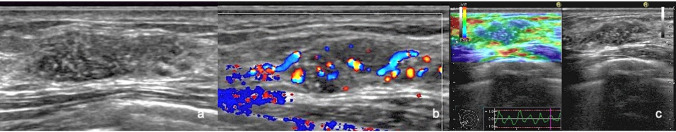
B-mode ultrasonography (US), color Doppler US, and elastography in a case of ductal carcinoma in situ. **a** A segmental hypoechoic area in the mammary gland with echogenic foci is seen on B-mode US. **b** It demonstrated vascularity of (3 +) on color Doppler US. **c** An elasticity score of 3 was assigned on elastography

**Fig. 6:**
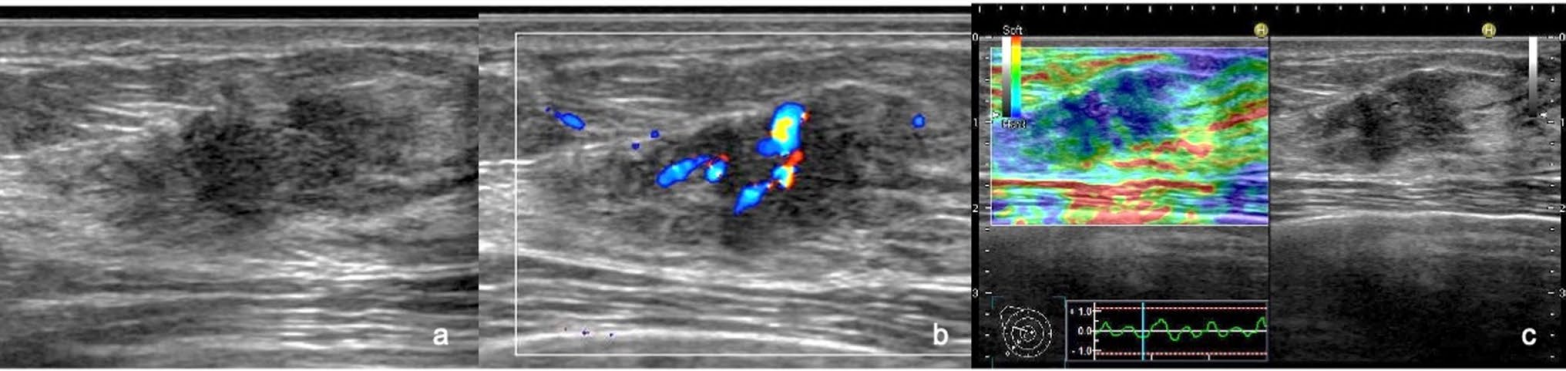
B-mode ultrasonography (US), color Doppler US, and elastography in a case of invasive lobular carcinoma. **a** A focal hypoechoic area in the mammary gland was seen on B-mode US. **b** It demonstrated vascularity of (2 +) on color Doppler US. **c** An elasticity score of 3 was assigned on elastography

**Fig. 7:**
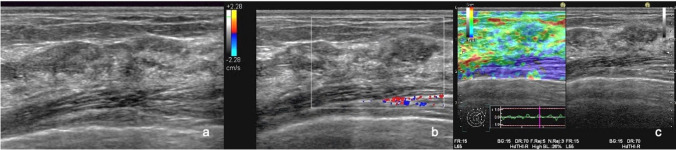
B-mode ultrasonography (US), color Doppler US, and elastography in a case of fibrocystic disease. **a** A regional hypoechoic area in the mammary gland was seen on B-mode US. **b** It demonstrated no vascularity on color Doppler US. **c** An elasticity score of 2 was assigned on elastography

Figure 5 shows a segmental hypoechoic area in the mammary gland with echogenic foci on B-mode US (Fig. 5a). Category 5 was assigned to the lesion. It demonstrated vascularity of (3 +) on color Doppler US (Fig. 5b), and an elasticity score of 3 was assigned on elastography (Fig. 5c). After surgery, the tumor was histologically diagnosed as ductal carcinoma in situ. Figure 6 shows a focal hypoechoic area in the mammary gland on B-mode US (Fig. 6a). Category 4 was assigned to the lesion. It demonstrated vascularity of (3 +) on color Doppler US (Fig. 6b), and an elasticity score of 3 was assigned on elastography (Fig. 6c). After surgery, the tumor was histologically diagnosed as invasive lobular carcinoma. Figure 7 shows a regional hypoechoic area in the mammary gland on B-mode US (Fig. 7a). Category 3b was assigned to the lesion. No vascularity was observed on color Doppler US (Fig. 7b), and an elasticity score of 2 was assigned on elastography (Fig. 7-c). On core needle biopsy, the lesion was histologically diagnosed as fibrocystic change.

### Diagnostic ability of color Doppler US and elastography adjunct to B-mode US

The addition of color Doppler US and SE to B-mode imaging in this study improved sensitivity without compromising specificity because in the case of invasive breast cancers, tumor cells infiltrating the stroma are accompanied by vascular hyperplasia, and in the case of noninvasive ductal carcinomas, the higher the degree of malignancy, the higher the vascular hyperplasia in the stroma, and the greater the vascularity (Figs. 5, 6) [23, 24]. Thus, color Doppler US is incomparable for the diagnosis of breast cancer with improved sensitivity. In contrast, benign lesions in the mammary gland are relatively soft and exhibit low scores on elastography, making it superior for diagnosing benign lesions, thus improving the specificity (Fig. 7) [3, 25]. Color Doppler and elastography work in a complementary manner, resulting in improved sensitivity while maintaining specificity.

For breast masses, the addition of color Doppler US and elastography to B-mode US has considerably improved specificity while maintaining sensitivity [14]. In this study, the specificity did not improve as much for non-mass abnormalities as for masses. This is because fibrocystic changes, which are benign lesions, often show a decrease in the elasticity, whereas noninvasive ductal carcinoma, which is a malignant lesion, often maintains its elasticity. Therefore, the addition of SE to B-mode and color Doppler US did not improve specificity and decreased sensitivity.

Vascularity of blood flow into an internal solid component of the ducts was not a statistically significant finding for distinguishing between 35 benign and 19 malignant lesions. Benign internal solid component of the duct included at least eight cases of intraductal papilloma, which has a relatively thick vascular stalk and exhibits abundant vascularity for its size. Therefore, we presume that no significant difference was present in the vascularity between the benign and malignant internal solid component of the ducts in this study.

### B-mode features and benign/malignant differential diagnosis

To differentiate between benign and malignant lesions, the JABTS guidelines assign categories based on B-mode distribution and spread of non-mass abnormalities, considering the presence of echogenic foci. However, statistically significant differences in the odds ratios were observed only in the presence of echogenic foci in the hypoechoic areas of the mammary gland and the configuration of the internal solid component in the mammary ducts. No difference was observed between benign and malignant non-mass abnormalities with respect to distribution and spread on B-mode US. The sensitivity of categories 3b, 4, and 5, which are indicated for tissue biopsy, as malignant tumors was 83.7%, and the specificity and accuracy were 32.8% and 59.5%, respectively, thereby indicating poor diagnostic ability. This is because benign lesions, such as epithelial hyperplasia, adenosis, and fibrosis, were classified as category 3b or higher, and the current category classification may be inappropriate for excluding benign lesions and indications for biopsies.

### Color Doppler US and elastography features and benign/malignant differential diagnosis

As described above, the diagnostic ability of B-mode ultrasound is insufficient for non-mass abnormalities, and we attempted to improve its diagnostic ability by incorporating color Doppler US and SE. The odds ratio of vascularity (2 +) and (3 +) versus (1 +) and (–) was 7.84, and that of penetrating blood flow was 7.46, which were considered significant findings for malignancy. Namely, vascularity evaluation using color Doppler US in this study was excellent for determining benign or malignant diseases, and it improved the sensitivity from 84.6%83.7% to 93.1%. It is relatively difficult to determine whether non-mass abnormalities are abnormalities or normal variations using B-mode images. In this sense, the increase in sensitivity resulting from adding color Doppler US to B-mode US in this study is considered meaningful. In contrast, the odds ratio of elasticity scores 3, 4, and 5 versus elasticity scores 1 and 2 was 4.49. The elastography findings in this study often overlapped between benign and malignant lesions, and the discrimination between benign and malignant lesions was inferior to that achieved using color Doppler US. Although no benign lesions with an elasticity score of 5 were detected, benign lesions with an elasticity score of 3 or 4 accounted for 50% of all lesions. Hence, adding elastography did not significantly improve the specificity.

### Distribution of vascularity by age between benign and malignant non-mass lesions

Patients with benign non-mass abnormalities aged under 55 years tended to have more lesions with high vascularity than those aged 55 years and older. We previously reported that vascularity was significantly higher in younger individuals under 50 years of age than in those over 50 years of age upon performing US examinations for benign breast masses using B-mode plus color Doppler US [10]. Even in benign breast diseases presenting with non-mass abnormalities, recognizing that vascularity of lesions can be higher in younger individuals suggests the potential for improving specificity.

### Future scope considering US of non-mass abnormalities of the breast.

Regarding B-mode US, no statistically significant difference was observed in the distribution and spread of lesions, which is thought to be the basic characteristic feature for differentiating between benign and malignant lesions. The accuracy rate was 59.5%, indicating that B-mode US alone could not differentiate benign and malignant tumors. Color Doppler US and SE were then added to B-mode US; although the sensitivity improved, the specificity did not change. Moreover, although the number of missed breast cancers could be reduced, they could not contribute to the avoidance of unnecessary biopsies.

Non-mass abnormalities of the mammary gland originally included lesions of various histological types, regardless of whether they were benign or malignant. Therefore, to diagnose non-mass abnormalities of the breast, it is necessary to comprehensively evaluate not only US but also clinical, mammography, and MRI findings to determine whether the tumor is benign or whether biopsy should be performed.

### Limitations

This study had several limitations. First, as mentioned earlier, non-mass abnormalities in the breast include a wide variety of histological types; therefore, an analysis for each subtype should be performed. Second, owing to their diversity, there are limitations to determining whether non-mass abnormalities are benign or malignant using uniform findings and criteria. Third, the cutoffs for vascularity on color Doppler US and the elasticity score on elastography have not been validated for determining whether a tumor is benign or malignant. Changing the cutoff automatically changes the sensitivity and specificity; therefore, setting an optimal cutoff is necessary. Finally, we considered the lesions to be benign during the 1-year follow-up. BI-RADS recommends 2-year follow-up, and we consider the 1-year follow-up to be insufficient. However, in actual clinical practice, there are few cases in which benign lesions can be followed up for 2 years. Therefore, if only those who have undergone strict follow-up observation for 2 years are considered eligible, many patients will be excluded.

## Conclusion

This study revealed that color Doppler US and SE are useful for differentiating malignant from benign non-mass abnormalities of the breast and improving the sensitivity while maintaining the specificity for detecting these lesions. However, the differential diagnosis of non-mass abnormalities of the breast using US is not satisfactory. Therefore, a comprehensive diagnosis, including mammography, MRI, and clinical findings, is indispensable.

## Supplementary Information

Below is the link to the electronic supplementary material.

Supplementary Table 1 Correspondence between Japanese and BI-RADS categories

Supplementary Fig. 1 Flowcharts of study sample selection

Supplementary Fig. 2 Distribution of vascularity by age between benign and malignant non-mass abnormalities

## Data Availability

All data generated or analyzed during this study are included in this published article.
