# Supplementary figures and images for: Usefulness of color Doppler and strain elastography adjunctive to B-mode ultrasonography in the diagnosis of non-mass abnormalities of the breast: results of the BC-07 multicenter study of 385 cases
Source: J Med Ultrason (2001). 2024 Aug 13;52(1):157–68. doi: 10.1007/s10396-024-01485-1 (PMC11799060; doi:10.1007/s10396-024-01485-1)

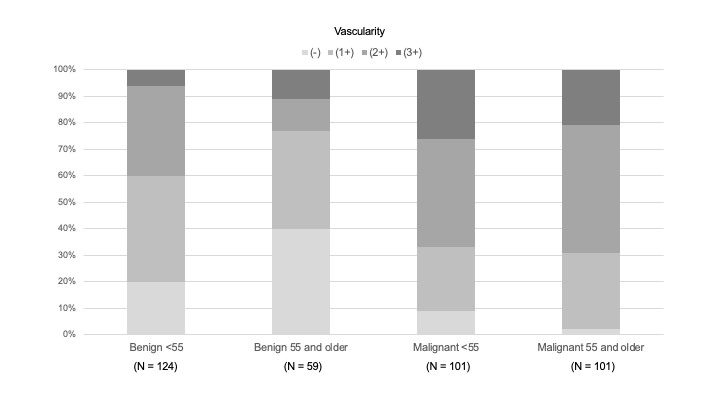

Supplement: Supplementary file 3 — Supplementary Fig. 2 Distribution of vascularity by age between benign and malignant non-mass abnormalities [file 10396_2024_1485_MOESM3_ESM.tiff]
